# *CLAVATA* modulates auxin homeostasis and transport to regulate stem cell identity and plant shape in a moss

**DOI:** 10.1111/nph.17969

**Published:** 2022-02-08

**Authors:** Zoe Nemec‐Venza, Connor Madden, Amy Stewart, Wei Liu, Ondřej Novák, Aleš Pěnčík, Andrew C. Cuming, Yasuko Kamisugi, C. Jill Harrison

**Affiliations:** ^1^ 1980 School of Biological Sciences University of Bristol 24 Tyndall Avenue Bristol BS8 1TQ UK; ^2^ Division of Psychological Medicine & Clinical Neurosciences MRC Centre for Neuropsychiatric Genetics & Genomics Cardiff University School of Medicine Heath Park Cardiff CF14 4XN UK; ^3^ Laboratory of Growth Regulators Faculty of Science of Palacký University and Institute of Experimental Botany of the Czech Academy of Sciences Šlechtitelů 27 Olomouc 78371 Czech Republic; ^4^ Centre for Plant Sciences Faculty of Biological Sciences University of Leeds Leeds LS2 9JT UK

**Keywords:** CLAVATA, CLV‐WUS, evo‐devo, moss filament identity, physcomitrella, plant stem cell

## Abstract

- The CLAVATA pathway is a key regulator of stem cell function in the multicellular shoot tips of Arabidopsis, where it acts via the WUSCHEL transcription factor to modulate hormone homeostasis. Broad‐scale evolutionary comparisons have shown that CLAVATA is a conserved regulator of land plant stem cell function, but CLAVATA acts independently of WUSCHEL‐like (WOX) proteins in bryophytes. The relationship between CLAVATA, hormone homeostasis and the evolution of land plant stem cell functions is unknown.
- Here we show that in the moss, Physcomitrella (*Physcomitrium patens*), CLAVATA affects stem cell activity by modulating hormone homeostasis. CLAVATA pathway genes are expressed in the tip cells of filamentous tissues, regulating cell identity, filament branching, plant spread and auxin synthesis. The receptor‐like kinase PpRPK2 plays the major role, and *Pprpk2* mutants have abnormal responses to cytokinin, auxin and auxin transport inhibition, and show reduced expression of *PIN* auxin transporters.
- We propose a model whereby *PpRPK2* modulates auxin gradients in filaments to determine stem cell identity and overall plant form.
- Our data indicate that CLAVATA‐mediated auxin homeostasis is a fundamental property of plant stem cell function, probably exhibited by the last shared common ancestor of land plants.

## Introduction

Organ size and shoot architecture are determined by the number and activity of stem cells in the growing shoot tips of flowering plants such as Arabidopsis (Fletcher, 2018). The size and integrity of Arabidopsis shoot tips is maintained by the action of a molecular feedback loop involving CLAVATA peptides and receptors and the WUSCHEL transcription factor (Schoof *et al*., 2000; Somssich *et al*., 2016). *CLAVATA3* is expressed in stem cells in the outermost cell layers (Fletcher *et al*., 1999), encoding a protein that is processed to form a small diffusible peptide (Rojo *et al*., 2002; Lenhard & Laux, 2003; Kondo *et al*., 2006; Ohyama *et al*., 2009). The CLAVATA3 peptide acts as a ligand to the CLAVATA1 receptor which is active in inner cell layers of the shoot tip (Clark *et al*., 1997; Ogawa *et al*., 2008), and signalling via CLAVATA1 confines the expression of *WUSCHEL* to a few cells at the centre of the shoot tip (Schoof *et al*., 2000). In turn, the WUSCHEL protein moves to the outermost cell layers of the shoot tip (Yadav *et al*., 2011; Daum *et al*., 2014), promoting expression of *CLAVATA3*. Mutants with defective CLAVATA or WUSCHEL function respectively over‐proliferate cells in the shoot tips or are unable to maintain the stem cell population, resulting in enlargement of the tips or shoot termination (Laux *et al*., 1996; Clark *et al*., 1997). CLAVATA function depends on the maintenance of low levels of auxin signalling permissive to stem cell activity in the central zone of the shoot tips by WUSCHEL (Schoof *et al*., 2000; Ma *et al*., 2019).

Unlike Arabidopsis, the growing tips of mosses such as *Physcomitrium patens* comprise a single apical cell (Harrison, 2017). Spores germinate to form a branching mat of filamentous tissue termed the protonema, and tip growth in the apical cells of each protonemal filament extend plant spread (Menand *et al*., 2007a). When growth commences, filaments have chloronemal identity and serve a primarily photosynthetic function, having many large dark green chloroplasts, but later on, more rapidly growing foraging filaments with smaller pale chloroplasts develop (caulonemata) (Ashton *et al*., 1979). Whereas chloronemal apical cells cleave in a plane perpendicular to the main axis of growth, caulonemal apical cells cleave obliquely and generate cells that are longer than chloronemal cells (Ashton *et al*., 1979). The relative growth of chloronemata and caulonemata determines the size and shape of the plant, such that plants comprising only chloronemata are small and round, whereas plants with predominantly caulonemata are larger and have an irregular foraging fringe (Prigge *et al*., 2010).

Auxin promotes caulonemal differentiation via a TRANSPORT INHIBITOR RESPONSE (TIR1)/AUXIN‐SIGNALING F‐BOX (AFB) and AUX/IAA‐dependent mechanism (Prigge *et al*., 2010; Viaene *et al*., 2014; Lavy *et al*., 2016; Thelander *et al*., 2018). Protonemal apical cells are the site of cell fate decisions that affect overall plant shape, yet auxin reporters suggest that they have minimal auxin sensing (Thelander *et al*., 2019). Exogenously applied cytokinin has a converse effect to auxin, suppressing caulonemal differentiation. Cytokinin promotes AUX/IAA expression, and cytokinin‐induced expression depends on auxin signalling (Ashton *et al*., 1979; Prigge *et al*., 2010). Thus, a complex interplay between auxin and cytokinin regulates protonemal tip cell identity, the chloronema‐to‐caulonema transition and overall plant shape.

The *P*. *patens* CLAVATA pathway comprises four CLAVATA3‐like peptides encoded with some redundancy by nine genes (*PpCLEs 1–9*), two CLAVATA1‐like receptors (PpCLV1a and PpCLV1b), and a further receptor similar to Arabidopsis receptor‐like kinase RPK2 (PpRPK2) (Goad *et al*., 2017; Fletcher, 2018; Whitewoods *et al*., 2018). Whilst *PpCLEs1–7* were identified from the V1.6 *Physcomitrium* genome, the sequences of *PpCLE8* and *PpCLE9* were identified using the V3 genome, and these genes encode the same peptides as *PpCLE1*, *PpCLE2* and *PpCLE3*, as shown in Table 1 (Whitewoods *et al*., 2018). Previous work demonstrated that *PpCLEs 1*, *2* and *7* and *PpCLV1a*, *PpCLV1b* and *PpRPK2* are highly expressed, and are required to establish stem cells that iterate shoot‐like structures (gametophores), affecting stem cell growth, identity and division plane orientations (Whitewoods *et al*., 2018). *PpcleAmiR1‐3*, *PpcleAmiR4‐7*, *Ppclv1a1b* and *Pprpk2* mutants had striking defects in gametophore development but comparatively normal protonemal development (Whitewoods *et al*., 2018).

**Table 1:** Peptide sequences encoded by *Physcomitrium patens* CLAVATA (CLV)/ENDOSPERM SURROUNDING REGION (ESR)‐related (CLE) genes.

Gene names	Peptide sequence encoded
*PpCLE1, PpCLE2* and *PpCLE3, PpCLE8* and *PpCLE9*	R **M** VP **T** GPNPLHN
*PpCLE4*	R **M** VP **S** GPNPLHN
*PpCLE5* and *PpCLE6*	R **L** VP **T** GPNPLHN
*PpCLE7*	R **V** VP **T** GPNPLHN

Residues colour‐coded in blue vary between peptides.

There are three *P. patens WUSCHEL*‐like homeobox (WOX) genes, and previous work detected constitutive expression of *PpWOX13LA* and *PpWOX13LB*, but no expression of *PpWOX13LC*; PpWOX13LA and PpWOX13LB protein fusions showed elevated expression in protonemal stem cells and stem cells forming in a leaf regeneration assay (Sakakibara *et al*., 2014). *Ppwox13lab* mutants were unable to initiate growth in leaf regeneration assays but were otherwise indistinguishable from wild‐type plants during gametophyte development, and this divergence in phenotypes suggests that *P. patens* WOX and CLAVATA genes act independently (Sakakibara *et al*., 2014). *Marchantia polymorpha* CLAVATA pathway components (*MpCLE2* and *MpCLV1*) likewise act in a WOX‐independent manner (Hirakawa *et al*., 2020). Thus, whilst CLAVATA is a conserved regulator of land plant stem cell function, WOX function appears inessential in bryophytes, raising questions about the evolution of the *CLAVATA*–*WUSCHEL* gene regulatory network and the regulation of stem cell function in bryophytes and the last shared common ancestor of land plants. Here we show that CLAVATA acts in protonemata to repress the chloronema to caulonema developmental transition and propose a model whereby CLAVATA regulates stem cell identity by modulating auxin homeostasis and PIN‐mediated auxin transport.

## Materials and Methods

### Plant growth

The *Physcomitrium patens* Gransden strain was used as the wild‐type (WT) for all experiments, and *PpcleAmiR1‐3*, *PpcleAmiR4‐7*, *Ppclv1a*, *Ppclv1b*, *Ppclv1a1b*, *Pprpk2*, *PpCLE1::NGG*, *PpCLE2::NGG*, *PpCLE7::NGG*, *PpCLV1A::NGG*, *PpCLV1B::NGG* and *PpRPK2::NGG* line generation strategies were described previously (Whitewoods *et al*., 2018) (CLV, CLAVATA; CLE, CLV/ENDOSPERM SURROUNDING REGION (ESR)‐related); RPK, receptor‐like kinase). *Ppclv1a1brpk2* mutants were a gift from Joe Cammarata, Adrienne Roeder and Mike Scanlon, and were generated by CRISPR‐Cas9 editing *PpRPK2* in a *Ppclv1a1b* mutant background (Cammarata *et al*., 2021). Plants were spot‐propagated on BCDAT media (0.5% Agar, 1 mM magnesium sulphate (MgSO_4_), 3.67 mM monopotassium phosphate (KH_2_PO_4_), 10 mM potassium nitrate (KNO_3_), 45 µM iron sulfate (FeSO_4_), 5 mM ammonium tartrate dibasic ((NH_4_)_2_C_4_H_4_O_6_), 0.5 mM CaCl_2_, 1 : 1000 dilution of Trace Elements Solution (0.614 mg l^−1^ H_3_BO_3_, 0.055 mg l^−1^ AlK(SO_4_)_2_.12H_2_O, 0.055 mg l^−1^ CuSO_4_.5H_2_O, 0.028 mg l^−1^ KBr, 0.028 mg l^−1^ LiCl, 0.389 mg l^−1^ MnCl_2_.4H_2_O, 0.055 mg l^−1^ CoCl_2_.6H_2_O, 0.055 mg l^−1^ ZnSO_4_.7H_2_O, 0.028 mg/LKI and 0.028 mg l^−1^ SnCl_2_.2H_2_O)) unless otherwise stated, and grown at 23°C in continuous light or at 22°C in long day conditions (16 h : 8 h, light : dark photperiod). Uniform 1 mm^2^ spots of 10–15‐d‐old filamentous tissue were used to inoculate growth experiments. To generate spores, protonemal homogenates were sown on peat plugs in Magenta vessels and grown at 23°C in continuous light for 8–10 wk before transfer to 16°C short day conditions (8 h : 16 h, light : dark). Mature sporogonia were harvested, incubated in 10% sodium hypochlorite for 5 min and washed three times with sterile water, then refrigerated or ruptured in sterile water and germinated in continuous light on BCDAT medium lacking Trace Elements Solution and with 10 mM CaCl_2_.

### Pharmacological treatments

1 mM and 100 µM 1‐naphthaleneacetic acid (NAA) and 100 µM and 10 µM 6‐Benzylaminopurine (BAP) stock solutions were prepared in 70% ethanol. 100 mM stocks of l‐Kynurenine (L‐Kyn) were prepared using dimethyl sulfoxide (DMSO) as a solvent. 5 mM *N*‐1‐naphthylphthalamic acid (NPA) stocks were prepared in 1 ml DMSO made up to 50 ml with 70% EtOH. All reagents were added to warm growth media before pouring plates.

### Phenotype analysis

Plant areas and perimeters were measured using fiji from images taken using a VHX‐1000 microscope (Keyence, Osaka, Japan) with a ×10 objective, excluding gametophores. These values were used to calculate the perimeter ratio, the ratio between the measured perimeter and the perimeter of a perfectly circular plant of the same area. For cell identity measurements, filaments protruding from the margins of 4‐wk‐old plants were dissected and stained with 0.3% Toluidine Blue for 2 min, rinsed in water and mounted on slides with coverslips before imaging with a DM2000 microscope (Leica, Wetzlar, Germany) using a ×40 objective or a VHX‐1000 microscope (Keyence) using a ×50–200 objective. The length and cell division angle of subapical cells of main filaments and of the second cell in branches with at least three cells were measured using fiji as described previously (Coudert *et al*., 2019).

### Generation of *promoter::NGG* lines

Promoter sequences from *PpCLE3* (2427 bp), *PpCLE4* (2867 bp), *PpCLE5* (1731 bp) and *PpCLE6* (1458 bp) were PCR‐amplified using a proofreading polymerase and cloned directly into the *Sma*I site of a modified PIG1NGGII (Ishikawa *et al*., 2011) vector in which an *NptII* resistance cassette was substituted for the *BSD* cassette (Whitewoods *et al*., 2018). The promoter plus the first few amino acids of the peptide coding sequence were PCR‐amplified from *PpCLE8* (3216 bp) and *PpCLE9* (1799 bp) before insertion into PIG1NGGII to generate a translational fusion with the reporter gene. All constructs were linearized with *Pme*I before plant transformation. Lines were screened using a forward primer from the *PIG1* targeting locus and a promoter‐specific reverse screening primer to check the 5′ integration site, and a CaMV terminator forward primer and reverse primer from the *PIG1* locus were used to check the 3′ integration site. Southern analyses to verify targeting were undertaken using either a green fluorescent protein (*GFP*)–β‐glucuronidase (*GUS*) probe PCR‐amplified to incorporate a DIG label (*PpCLE3*, *PpCLE4*, *PpCLE5*, *PpCLE6* and *PpCLE9*) or a probe against the *35S::NptII* resistance cassette (*PpCLE8*) as illustrated in Supporting Information Fig. S1, and using methods described in Whitewoods *et al*., (2018). Primer sequences are listed in Table S1.

### Nucleic acid extraction

DNA for PCR and Southern analysis was extracted using a modified CTAB protocol (Doyle & Doyle, 1990). RNA for expression analyses was extracted from 5 d‐old protonemata using a RNeasy Plant Mini Kit (Qiagen). Genomic DNA removal and cDNA synthesis were performed with a Quantitect Reverse Transcription kit (Qiagen).

### Southern analysis

Southern blot analysis was carried out as described previously (Kamisugi *et al*., 2005). Genomic DNA (2.5 μg) was digested with *Hin*dIII (*PpCLE3*, *PpCLE4*, *PpCLE5*, *PpCLE8* and *PpCLE9*) or *Sca*I (*PpCLE6*), and transferred to HyBond‐N‐Plus membrane for hybridization with the digoxygenin‐labelled GFP–GUS reporter sequence.

### Expression analysis

Reverse transcription (RT)‐PCR was performed using a 1 : 10 dilution of cDNA as a template, and EcoTaq polymerase (Desai & Pfaffle, 1995). Where possible, primers were designed to span an intron or were placed on an intron–exon boundary, and a *UBIQUITIN* transcript was amplified as a positive control (Table S2). Quantitative (q)PCR was performed using a SYBR Green Quantitect kit and a Stratagene M×3005P bioanalyser with 95°C for 15 min, and then 94°C for 15 s, 60°C for 30 s, 72°C for 30 s cycling conditions for 40 cycles. Amplicon size was checked by dissociation curve. The efficiency of each primer pair was calculated on serial dilutions of WT cDNA, and only primer pairs with an efficiency between 90% and 110% were used in further experiments. To calculate fold‐change for each sample relative to the WT expression levels, the ΔΔCt method was used (Livak & Schmittgen, 2001). *PINA*, *PINB*, *PINC*, *PIND* and *60S* genes were amplified using primers listed in Table S3.

### Histochemical expression analysis

**Fig. 1:**
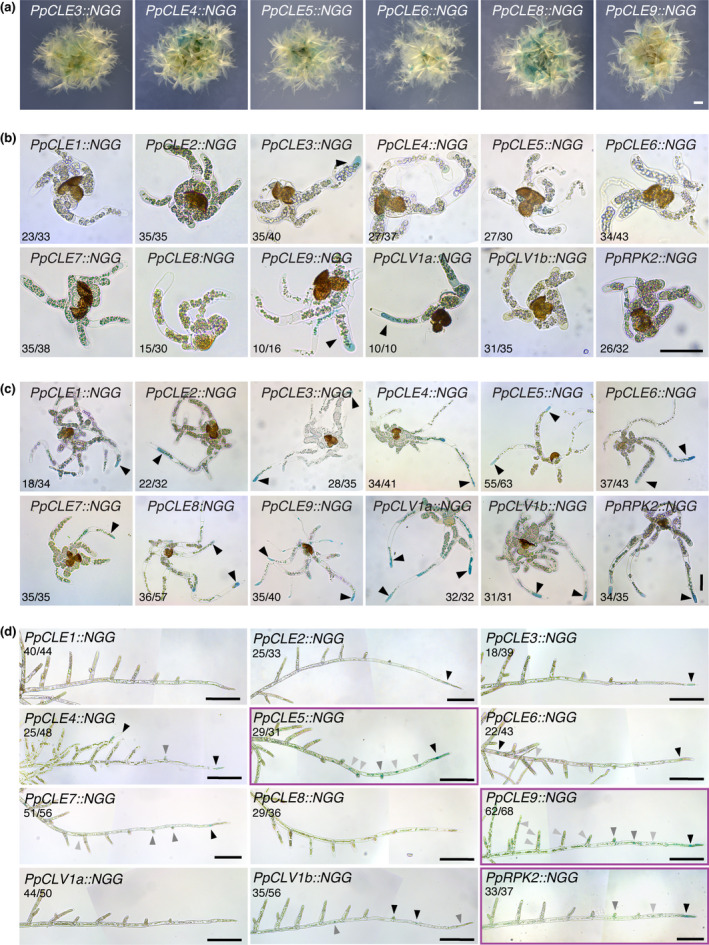
Caulonemal tip cells are likely sites of CLAVATA (CLV)/ENDOSPERM SURROUNDING REGION (ESR)‐related (CLE) peptide production and perception in *Physcomitrium patens*. (a) Micrographs showing expression patterns of newly generated *PpCLE::NGG* lines in whole plants, with signal in gametophores and protonemal tissues. Bar, 1 mm. (b) Micrographs of sporelings which comprised mainly chloronemata. *PpCLE3::NGG*, *PpCLE9::NGG* and *PpCLV1a::NGG* lines showed expression. The numbers in each panel indicate the proportion of sporelings displaying a similar expression pattern. Bar, 50 µm. (c) Micrographs of sporelings comprising a mix of chloronemata and caulonemata. All lines showed *CLAVATA* expression. The signal was absent or weak in chloronemal cells, but stronger and more frequently detected in caulonemal tip cells (black arrowheads). Numbers in each panel indicate the proportion of sporelings displaying a similar expression pattern. Bar, 50 µm. (d) Stitched light micrographs showing *promoter::NGG* reporter expression in foraging filaments. Although no signal was detected in the majority of *PpCLE1::NGG*, *PpCLE8::NGG* and *PpCLV1a::NGG* filaments, all other lines accumulated signal in caulonemal tip cells. Stain also was detected in a subset of lines in other caulonemal cells (*PpCLE4::NGG*, *PpCLE5::NGG*, *PpCLE6::NGG*, *PpCLE9::NGG*, *PpCLV1b::NGG*, *PpRPK2::NGG*), in new branch initials (*PpCLE5::NGG*, *PpCLE7::NGG*, *PpCLE9::NGG*, *PpCLV1b::NGG*, *PpRPK2::NGG*) or in chloronemal branch cells (*PpCLE4::NGG*, *PpCLE9::NGG*) (RPK, RECEPTOR‐LIKE KINASE). Both *PpCLV1b::NGG* and *PpRPK2::NGG* accumulated stain in caulonemal tip cells and new branch/bud initials but *PpRPK2::NGG* stained more strongly in caulonemal tip cells, and a subset of samples showed only this signal (4 of 37), whereas a subset of *PpCLV1B::NGG* samples accumulated signal only in the branch initials (12 of 56). Numbers in each panel indicate the proportion of filaments displaying a similar expression pattern. Tissue was stained for 7.5 h (*PpCLE3::NGG*, *PpCLE4::NGG*, *PpCLE5::NGG*, *PpCLE6::NGG*, *PpCLE8::NGG*, *PpCLV1b::NGG* and *PpRPK2::NGG*), 15 h (*PpCLE2::NGG*, *PpCLE9::NGG* and *PpCLV1a::NGG*) or 21 h (*PpCLE1::NGG* and *PpCLE7::NGG*). Black arrowheads indicate the strongest expression, grey arrows indicate weaker expression. Purple frames indicate lines with strong expression in caulonemal tip cells. Bar, 200 µm.

For whole plant expression analyses, plants were grown on BCDAT media containing 0.5% agar, cut out of plates and immersed in a staining solution which comprised 100 mM phosphate buffer (pH 7.0), 10 mM Tris‐HCl (pH 8.0), 1 mM ethylenediaminetetraacetic acid (EDTA), 0.05% Triton X‐100, 2 mM potassium ferricyanide, 2 mM potassium ferrocyanide and 1 mg ml^−1^ X‐Gluc (5‐bromo‐4‐chloro‐3‐indolyl‐β‐d‐glucuronic acid) dissolved in 10% (v/v) DMSO). Samples were incubated at 37°C for 7.5 h (*PpCLE3::NGG*, *PpCLE4::NGG*, *PpCLE5::NGG*, *PpCLE6::NGG*, *PpCLV1b::NGG* and *PpRPK2::NGG*), 15 h (*PpCLE2::NGG*, *PpCLE8::NGG*, *PpCLE9::NGG* and *PpCLV1a::NGG*) or 21 h (*PpCLE1::NGG* and *PpCLE7::NGG*) except for samples in Fig. 1(a), which were incubated for a third of the time with 0.5 mM potassium ferricyanide and 0.5 mM potassium ferrocyanide instead. Reactions were stopped by substituting the staining solution with ethanol. Samples were bleached overnight in 70% ethanol, rinsed in water then imaged using a Keyence VHX‐1000 digital microscope (whole plants) or a Leica DM2000 microscope (sporelings and filaments). For spore and sporeling expression analyses, plants were germinated on cellophane discs, moved to staining solution and imaged immediately after incubation.

### Hormone quantification

Hormone quantification by an ultra‐high performance liquid chromatography‐electrospray tandem mass spectrometry (UHPLC‐MS/MS) was undertaken using protonemal homogenates grown through three passages of 5 d in continuous light. Fifty milligrams of tissue were snap‐frozen in liquid nitrogen, and hormone extraction and quantification were undertaken as described by Novák *et al*., (2012) and Svaçinová *et al*. (2012).

## Results

### *P. patens CLAVATA* genes are expressed in protonemata

In order to investigate roles for CLAVATA in protonemal development, we first engineered *promoter::NLSGFPGUS* (*promoter::NGG*) reporter lines for *PpCLE3*, *PpCLE4*, *PpCLE5*, *PpCLE6*, *PpCLE8* and *PpCLE9* whose expression was uncharacterized in previous work (Whitewoods *et al*., 2018) (Fig. 1a, S1). Analysis of 3‐ to 4‐wk‐old plants revealed signal in protonemal tissues and gametophores for all *promoter::NGG* lines, and expression patterns were consistent between independently generated lines (not shown). To determine when *CLAVATA* expression first arises during development, spores from previously established *PpCLE1::NGG*, *PpCLE2::NGG*, *PpCLE7::NGG*, *PpCLV1a::NGG*, *PpCLV1b::NGG* and *PpRPK2::NGG* lines (Whitewoods *et al*., 2018), and newly engineered *PpCLE3::NGG*, *PpCLE4::NGG*, *PpCLE5::NGG*, *PpCLE6::NGG*, *PpCLE8::NGG* and *PpCLE9::NGG* lines were germinated and grown for 2–11 d before staining (Fig. 1b,c). *PpCLV1a::NGG* signal was detected at germination (Fig. S2), and *PpCLE3::NGG*, *PpCLE9::NGG* and *PpCLV1a::NGG* signal was detected in primary chloronemata (Fig. 1b). Following caulonemal differentiation, all lines showed signal in caulonemal tip cells (Fig. 1c).

Caulonemal filaments extending from the foraging fringe were dissected to observe expression at later developmental stages (Fig. 1d). No signal was detected in *PpCLE1::NGG*, *PpCLE8::NGG* or *PpCLV1a::NGG* lines, but the promoters of all remaining *PpCLEs* were active in caulonemal tip cells, and *PpCLE5::NGG* and *PpCLE9::NGG* lines showed the strongest signal. *PpCLV1b::NGG* lines frequently showed signal in side branch initials and the second and third cells from the tip, and *PpRPK2::NGG* lines showed strong signal in the majority of caulonemal tip cells. Taken together these expression data indicate roles for *PpCLE5*, *PpCLE9* and *PpRPK2* in CLE production and perception in caulonemal tip cells, and to a lesser extent indicate potential roles for *PpCLV1b* in caulonemal tip cells and branch initials.

### *P. patens clavata* mutants have cell identity and plant shape defects

**Fig. 2:**
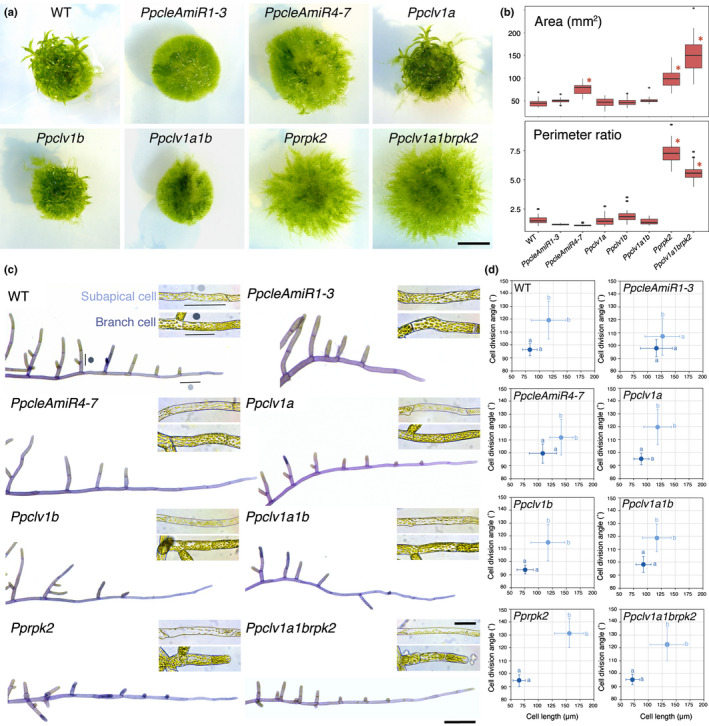
CLAVATA pathway components regulate plant shape and protonemal identity in *Physcomitrium patens*. (a) Images of 4‐wk‐old plants grown in continuous light showing overall morphology. Bar, 5 mm. (b) Plant spread in *PpcleAmiR4‐7*, *Pprpk2* and *Ppclv1a1brpk2* mutants was greater than wild‐type (WT) plant spread, and triple mutants were larger than *Pprpk2* mutants (CLE, CLAVATA (CLV)/ENDOSPERM SURROUNDING REGION (ESR)‐related; RPK, RECEPTOR‐LIKE KINASE). *Pprpk2* and *Ppclv1a1brpk2* mutants had a higher perimeter ratio than WT plants. Data from one of three experimental replicates are shown (*n* = 32). In the boxplot, horizontal lines represent median values, boxes represent the interquartile range, whiskers represent largest and smallest values within 1.5× above or below 75^th^ and 25^th^ percentiles, respectively, and black circles represent outliers. Asterisks indicate significant difference from the WT (one‐way ANOVA and Tukey’s honestly significant difference test; *P* < 0.05). (c) Light micrographs of toluidine blue stained and dissected protonemal filaments, showing differences in apical dominance and cell morphology between genetic backgrounds. Insets show typical unstained subapical and branch cells from each line. Bars: 200 µm (main); 50 µm (inset). (d) Subapical cells (pale blue dots; caulonemal cells in WT) and branch cells (dark blue dots; chloronemal cells in WT) have distinct shapes in Wt plants, *PpcleAmiR4‐7, Ppclv1a*, *Ppclv1b*, *Ppclv1a1b*, *Pprpk2* and *Ppclv1a1brpk2* mutants, but *PpcleAmiR1‐3* mutant cell types are less distinct. Subapical cell and branch cell measurements partially overlap in all lines except *Pprpk2*. Different letters indicate significant difference in cell length or division plane orientation, bars indicate SD (*n* ≥ 28; two‐way ANOVA *P* < 0.05).

In order to investigate roles for CLAVATA in protonemal development, we first quantified overall plant spread in WT and mutant plants (Fig. 2a,b). We also quantified a measure of plant shape, the perimeter ratio, which reflects the circularity of plant spread. A perimeter ratio of 1 corresponds to a perfectly circular shape, and higher values indicate irregular plant shapes with no increase in area and increased production of caulonemata (Fig. 2b). Whilst *PpcleAmiR1‐3* mutants had a similar size and shape to WT plants, *PpcleAmiR4‐7* mutants had greater spread but similar perimeter ratios to the WT, implying increased but uniform protonemal growth (Fig. 2b). Amongst receptor mutants, *Ppclv1a*, *Ppclv1b* and *Ppclv1a1b* mutants sometimes had increased areas and perimeter ratios, but the size and shape differences from WT plants were subtle and variable between experimental replicates. By contrast, *Pprpk2* and *Ppclv1a1brpk2* mutants were consistently and significantly larger with higher perimeter ratios than WT plants, indicating higher caulonema production and a key role for *PpRPK2* in plant size and shape determination (Fig. 2b).

In order to further investigate the cellular basis of plant spread and perimeter ratio differences between lines, protonemal morphology was quantified by measuring the length and cell division plane orientations of subapical cells of main filaments (subapical cells) and side branches (branch cells), as these report differences between caulonemal and chloronemal identity (Coudert *et al*., 2019) (Fig. 2c,d). *Ppclv1a*, *Ppclv1b* and *Ppclv1a1b* mutants showed no significant differences from WT plants with respect to subapical and branch cell length or cell division plane orientation in most experimental replicates (Fig. S3). However, although *PpcleAmiR1‐3* mutants were the same overall size as wild‐type plants, their cell types showed mixed chloronemal and caulonemal identities, with less oblique cell divisions in subapical cells and longer branch cells than WT plants (Fig. S3). *PpcleAmiR4‐7* mutant protonemata retained distinct cell identities but had longer subapical and branch cells than WT plants. *Pprpk2* and sometimes *Ppclv1a1brpk2* mutants had more distinct cell identities than WT plants, with longer and more oblique cell division plane orientations (Fig. S3).

More subtle differences in protonemal morphology also were observed following filament dissection (Fig. 2c). Wild‐type filaments initiate branches from the second subapical cell, and branch growth continues to give protonemata regular branching patterns (Fig. 2c). Branches close to the tip were longer in *PpcleAmiR1‐3* mutants than WT branches in equivalent positions, and *Pprpk2* and *Ppclv1a1brpk2* mutants conversely had shorter branches (Fig. 2c, S4). Thus, CLE signalling regulates protonemal morphology in *P*. *patens* and *PpRPK2* holds the main role as a repressor of plant spread, perimeter ratio and caulonemal identity.

### *Pprpk2* mutants are hypersensitive to cytokinin application

As *Ppclv1a* and *Ppclv1b* mutant phenotypes were variable, and *PpcleAmiR* mutant phenotypes reflect changes in expression of several *PpCLE*s (Fig. S5), we focused further functional analyses on *Ppclv1a1b*, *Pprpk2* and *Ppclv1a1brpk2* mutants. In WT plants, filament identity reflects a hormonal interplay between auxin and cytokinin, and cytokinin suppresses caulonemal identity (Ashton *et al*., 1979). We therefore reasoned that *PpRPK2* could promote cytokinin biosynthesis or enhance plants' response to cytokinin. To test the hypothesis that PpRPK2 promotes cytokinin biosynthesis, we used LC‐MS/MS to quantify cytokinin concentrations in protonemal tissue from *Ppclv1a1b*, *Pprpk2* and *Ppclv1a1brpk2* mutants and WT controls. In flowering plants, cytokinin bases, and to a lesser extent ribosides are the active forms, whereas nucleotides and *O*‐glucosides (OG) are inactive and can function in storage (Kieber & Schaller, 2018). In *P. patens* isopentenyl adenine (iP), *trans*‐zeatin (*t*Z) and their corresponding ribosides were shown to have the highest biological activity in a bud induction assay (von Schwartzenberg *et al*., 2007), whereas neither *cis*‐zeatin (*c*Z), *cis*‐zeatin riboside (*c*ZR) nor any of the ribotides had a bud inductive role. Twenty‐six types of cytokinin were assayed, including free bases as iP, *t*Z, *c*Z and dihydrozeatin (DHZ), and their ribotide (MP), riboside (R) and glycoside derivatives (Fig. S6). Thirteen types of cytokinins were detected, and although some mutant‐specific differences were present at the level of conjugates such as *c*ZRMP, *t*ZOG and *t*ZROG, the only free base present at higher concentrations in mutants was *c*Z in *Ppclv1a1b* mutants. No difference in the overall level of cytokinin was detected. Thus, the hypothesis that PpRPK2 reduces plant spread by promoting cytokinin biosynthesis was rejected.

**Fig. 3:**
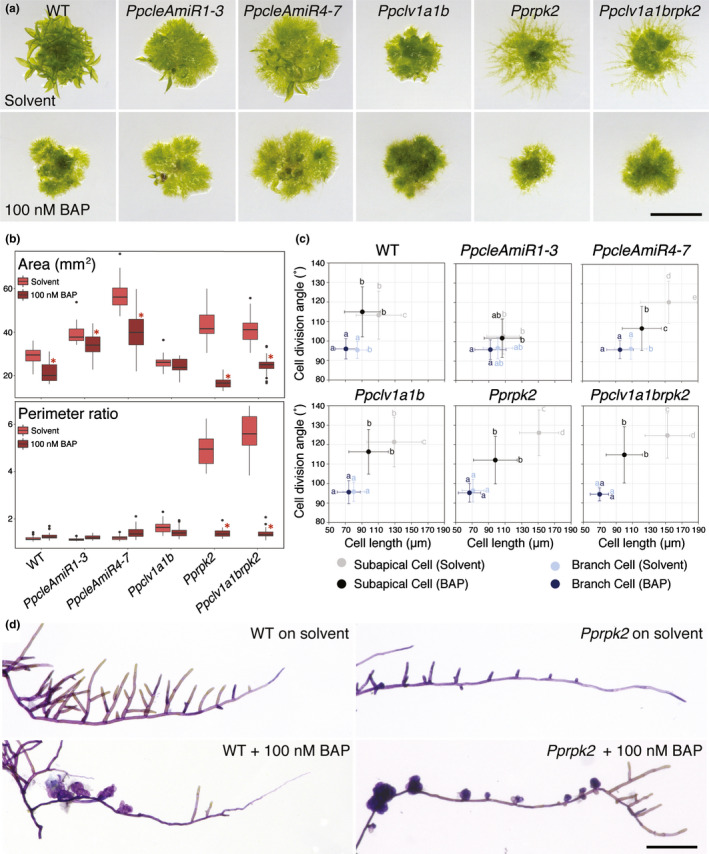
Cytokinin treatment disproportionally affects protonemal growth in *Physcomitrium patens clavata* mutants. (a) Micrographs of 4‐wk‐old plants grown on media containing a solvent control (EtOH) or cytokinin (100 nM 6‐Benzylaminopurine (BAP)). Bar, 5 mm. (b) Whilst *Ppclv1a1b* plant area was not significantly affected by cytokinin treatment, wild‐type, (WT) *PpcleAmiR1‐3*, *PpcleAmiR4‐7*, *Pprpk2* and *Ppclv1a1brpk2* plants showed decreased area, with the decrease being more conspicuous in *Pprpk2* and *Ppclv1a1brpk2* mutants (CLV, CLAVATA; (CLE, CLAVATA (CLV)/ENDOSPERM SURROUNDING REGION (ESR)‐related; RPK, RECEPTOR‐LIKE KINASE). The perimeter ratio did not change in response to cytokinin treatment in WT, *PpcleAmiR1‐3*, *PpcleAmiR4‐7* or *Ppclv1a1b* plants, but strongly decreased in *Pprpk2* and *Ppclv1a1brpk2* mutants. In the boxplot, horizontal lines represent median values, boxes represent the interquartile range, whiskers represent largest and smallest values within 1.5× above or below 75^th^ and 25^th^ percentiles, respectively, and black circles represent outliers. Asterisks indicate significant difference to EtOH control (multi‐way ANOVA and Tukey's honestly significant difference (HSD) test; *P* < 0.05; *n* ≥ 30). (c) Graphs showing a reduction in subapical cell length in response to cytokinin application in WT and *Ppclv1a1b* plants, and a strong reduction in subapical cell length and division plane angles in *PpcleAmiR4‐7*, *Pprpk2* and *Ppclv1a1brpk2* mutants grown on 100 nM BAP. Letters indicate significant differences between groups, error bars indicate SD (*n* ≥ 90; multi‐way ANOVA and Tukey's HSD test; *P* < 0.05). (d) Micrographs of caulonemal filaments dissected from WT or *Pprpk2* mutants grown on plates containing a solvent control (EtOH) or 100 nM BAP. *Pprpk2* mutants treated with BAP produced a near constitutive overbudding phenotype. Bar, 200 µm.

We next tested the hypothesis that PpRPK2 enhances plants' response to cytokinin. To this end, we grew mutants and WT plants on media containing a solvent control (0.07% EtOH) or 100 nM of the synthetic cytokinin 6‐benzylaminopurine (BAP), and analyzed their phenotypes after 4–5 wk of growth (Fig. 3). As expected, WT plants showed a decrease in plant spread in response to exogenously applied BAP (Fig. 3a,b). On average, the area of WT plants grown on 100 nM BAP was 26% ± 11% smaller than that of control plants, with no difference in perimeter ratio. The response to BAP treatment was sometimes weaker in *PpcleAmiR1‐3* and *Ppclv1a1b* mutants than in controls, but *Pprpk2* and *Ppclv1a1brpk2* plants grown on 100 nM BAP consistently showed an enhanced response, with a 59% ± 3% and 43% ± 9% (respectively) decrease in plant spread and reductions in perimeter ratio (Fig. 3a,b).

In order to study cytokinin responses at the cellular level, we dissected foraging filaments from plants and measured cell lengths and division planes as described previously (Fig. 3c). In WT plants, BAP treatment reduced cell lengths in both subapical and branch cells, whereas division plane angles were unaffected. In *Ppclv1a1b* and *PpcleAmiR1‐3* mutant subapical cells, responses to cytokinin were respectively similar to or weaker than WT responses. However, *PpcleAmiR4‐7*, *Pprpk2* and *Ppclv1a1brpk2* subapical cells showed a large reduction in both cell length and division plane angle following BAP treatment. Thus, *Pprpk2* and *Ppclv1a1brpk2* mutant plants have an enhanced response to cytokinin in caulonemal tip cells as well as in whole plants, refuting the hypothesis that *PpRPK2* promotes plants' response to cytokinin. *Pprpk2* mutants showed further evidence of cytokinin hypersensitivity in gametophore initiation, which is normally upregulated by cytokinin (Ashton *et al*., 1979). Whilst WT plants grown on BAP showed a higher frequency of gametophore initiation than untreated controls, *Pprpk2* mutants grown on BAP showed almost constitutive gametophore initiation (Fig. 3d).

### Auxin synthesis is not elevated in *Pprpk2* mutants, but mutant increases in plant spread require auxin

**Fig. 4:**
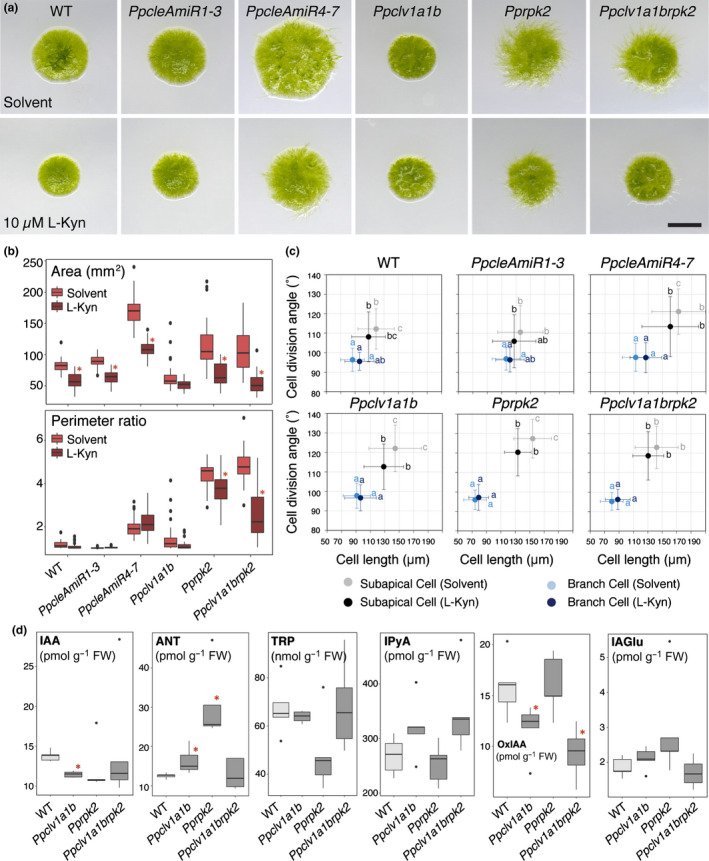
*Physcomitrium patens rpk2* and *clv1a1brpk2* mutant phenotypes depend on auxin synthesis, and mutants have synthesis defects (CLV, CLAVATA; RPK, RECEPTOR‐LIKE KINASE). (a) Micrographs of 4‐wk‐old plants grown on media containing a solvent control (DMSO) or a pharmacological inhibitor of auxin synthesis (10 µM l‐Kynurenine (L‐Kyn)). Bar, 5 mm. (b) Plant spread in all lines diminishes in response to L‐Kyn, but perimeter ratio values decreased only in *Pprpk2* and *Ppclv1a1brpk2* mutants (two‐way ANOVA and Tukey's honestly significant difference (HSD) test; *n* ≥ 30; *, *P* < 0.05 between treatment and control). (c) L‐Kyn treatment suppresses subapical cell length in *Ppclv1a1b* and *Pprpk2* mutants, and suppresses oblique cell divisions in *PpAmiR4‐7*, *Ppclv1a1b* and *Pprpk2* mutants. Letters indicate significant differences between groups, error bars indicate SD (multi‐way ANOVA and Tukey's HSD test, *n* ≥ 90, *P* < 0.05). (d) Quantification of auxin metabolites from wild‐type, *Ppclv1a1b*, *Pprpk2* and *Ppclv1a1brpk2* protonemal cultures showed that auxin levels were depleted in *Ppclv1a1b* and *Pprpk2* mutants in 4 of 5 biological replicates. IAA, Indole‐3‐acetic acid; ANT, Anthranilate; TRP, Tryptophan; IPyA, Indole‐3‐pyruvic acid; OxIAA, 2‐oxindole‐3‐acetic acid; IAGlu, IAA‐glucose; Student’s *T*‐test; *n* = 5; *, *P* < 0.05.

Because auxin can enhance or suppress cytokinin activity in *P. patens* (Ashton *et al*., 1979), we hypothesized that cytokinin hyper‐sensitivity in *Pprpk2* and *Ppclv1a1brpk2* mutants could reflect differences in auxin biosynthesis. To test the dependence of *clavata* mutant phenotypes on auxin biosynthesis, we grew WT and mutant plants on media containing a pharmacological inhibitor of auxin synthesis (10 µM L‐Kyn) or a solvent control (0.01% DMSO) (Fig. 4a‐c). When grown on L‐Kyn, WT plants showed significantly smaller areas than plants grown on a solvent control. Area decreased in all *clavata* mutants except *Ppclv1b*, and decreases were largest in *PpcleAmiR4‐7*, *Pprpk2* and *Ppclv1a1brpk2* mutants (Fig. 4b). Perimeter ratios were consistently unaffected by L‐Kyn in WT, *PpcleAmiR1‐3*, *PpcleAmiR4‐7* and *Ppclv1a1b* plants. Whilst *Pprpk2* and *Ppclv1a1brpk2* mutant perimeter ratios remained higher than WT values, perimeter ratios decreased significantly following L‐Kyn treatment in these mutants, indicating that their irregular shapes depend on auxin synthesis. To test the effect of L‐Kyn on cell identity, we dissected foraging filaments and measured cell length and cell division plane orientations. While no response to L‐Kyn was detected in branch cells, subapical cells of all lines were shorter and/or had division plane angles closer to 90° when plants were grown on L‐Kyn, and these differences were significant in *PpcleAmiR4‐7*, *Ppclv1a1b* and *Pprpk2* mutants. Hence, WT and *clavata* mutant plants have a qualitatively similar response to auxin biosynthesis inhibition, and auxin synthesis is needed for the caulonemal overproliferation phenotype of *Pprpk2* and *Ppclv1a1brpk2* mutants.

As auxin promotes caulonemal development, we hypothesized that higher levels of auxin production could account for *Pprpk2* and *Ppclv1a1brpk2* mutant phenotypes. We therefore quantified biologically active auxin IAA, and its precursors anthranilate (ANT); l‐tryptophan (TRP); and indole‐3‐pyruvic acid (IPyA) and degradation products IAA‐glutamate (IAGlu) and 2‐oxindole‐3‐acetic acid (OxIAA) in protonemal tissues of WT and mutant plants. However, we found that *Ppclv1a1b* and four of five replicates of *Pprpk2* tissue batches in fact had lower IAA concentrations than WT protonemata, whereas *Ppclv1a1brpk2* IAA concentrations were variable (Fig. 4d). *Ppclv1a1b* and *Pprpk2* protonemata contained more ANT than WT protonemata, and *Ppclv1a1b* and *Ppclv1a1brpk2* mutant protonemata contained less OxIAA than WT samples. As *Ppclv1a1b* (and usually *Pprpk2* mutant) protonemata contained less biologically active auxin (IAA) than WT protonemata, we rejected our hypothesis that higher levels of auxin synthesis contribute to *Pprpk2* and *Ppclv1a1brpk2* mutant phenotypes.

### *Pprpk2* mutants show abnormal developmental responses to auxin

**Fig. 5:**
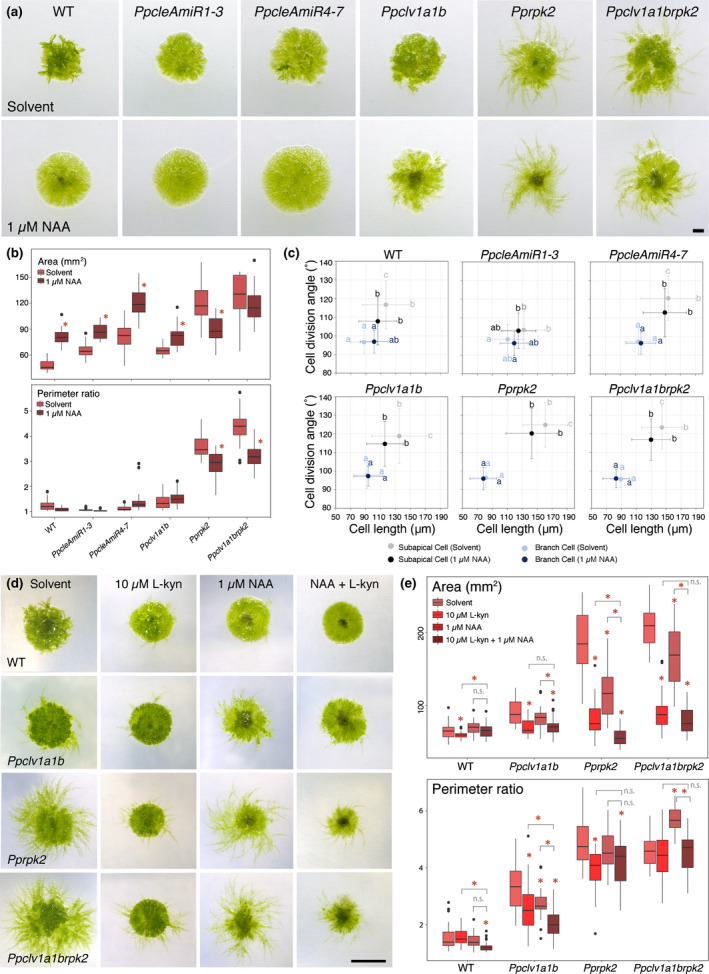
*Physcomitrium patens rpk2* and *clv1a1brpk2* mutants manifest an abnormal auxin response (CLV, CLAVATA; RPK, RECEPTOR‐LIKE KINASE). (a) Micrographs of 4‐wk‐old plants grown on media containing a solvent control (EtOH) or auxin (1 µM 1‐naphthaleneacetic acid (NAA)). Bar, 1 mm. (b) Quantitative analyses showed that *Pprpk2* and *Ppclv1a1brpk2* mutant plants show an auxin‐dependent decrease in plant spread and perimeter ratio, whilst all other backgrounds increased in area in response to auxin treatment and showed no change in perimeter ratio (two‐way ANOVA and Tukey's honestly significant difference (HSD) test. *n* ≥ 30; *, *P* < 0.05 between treatment and control). (c) Wild‐type (WT), *PpcleAmiR4‐7* and *Ppclv1a1brpk2* subapical cell division angles diminished following 1 µM NAA treatment, and *Ppclv1a1b* and *Pprpk2* subapical cell lengths decreased, but branch cells and *PpcleAmiR1‐3* mutant cells showed no change in length or cell division plane angle. Letters indicate significant differences between groups, error bars indicate SD (multi‐way ANOVA and Tukey's HSD test; *n* ≥ 90 for all other genotypes; *P* < 0.05). (d) Micrographs of 4‐wk‐old plants grown on media containing a solvent control (0.07% EtOH + 0.01% DMSO), 10 µM l‐Kynurenine (L‐Kyn), 1 µM NAA or a combination of 10 µM L‐Kyn and 1 µM NAA. Bar, 5 mm. (e) Quantitative analyses showed that whilst WT plant spread showed little response to exogenously applied auxin (1 µM NAA), auxin synthesis inhibitors (10 µM L‐Kyn) or a combination of 1 µM NAA and 10 µM L‐Kyn, *Pprpk2* mutant spread strongly decreased in all treatments and the combined treatment led to the strongest decrease. The response of *Ppclv1a1b* and *Ppclv1a1brpk2* mutants varied between experimental replicates, as did perimeter ratios. Asterisks above data indicate significant difference from untreated controls, asterisks above bars indicate significant difference between treatments of interest (*n* ≥ 30; one‐way ANOVA and Tukey’s HSD test on each genotype; *P* < 0.05). In boxplots, horizontal lines represent median values, boxes represent the interquartile range, whiskers represent largest and smallest values within 1.5× above or below 75^th^ and 25^th^ percentiles, respectively, and black circles represent outliers.

Enhanced sensitivity to auxin could yield similar developmental outcomes to high auxin concentrations. To evaluate the response of *clavata* mutants to exogenous auxin, we grew plants on media containing either the synthetic auxin 1‐naphthaleneacetic acid (1 µM NAA) or a solvent control (0.07% EtOH) (Fig. 5a‐c). In line with previous studies (Ashton *et al*., 1979; Lavy *et al*., 2016), WT plant areas increased when grown with additional auxin, but perimeter ratios were unaffected. Although *PpcleAmiR1‐3*, *PpcleAmiR4‐7* and *Ppclv1a1b* plants showed the same response as WT plants, *Pprpk2* plants conversely showed both area and perimeter ratio decreases (Fig. 5a,b). At the cellular level, WT subapical cells showed a strong response to NAA application with division angles close to 90°. *PpcleAmiR4‐7* and *Ppclv1a1brpk2* mutants also showed significant reductions in division angle following auxin application, and *Ppclv1a1b* and *Pprpk2* mutants showed significant reduction in subapical cell lengths.

In order to further dissect the effects of auxin synthesis and auxin response, we uncoupled these two processes by growing plants on media containing either a solvent control (0.01% DMSO + 0.07% EtOH), 10 µM L‐Kyn (reduced synthesis), 1 µM NAA (response plus endogenous synthesis), or a combination of 10 µM L‐Kyn and 1 µM NAA (response in absence of synthesis). We reasoned that if mutant phenotypes are caused by enhanced auxin perception or response, mutants would respond in a similar way to saturating concentrations of exogenously applied auxin (1 µM NAA treatment) both with and without endogenous synthesis (combined treatment). Wild‐type plant spread increased in L‐Kyn + NAA treated plants relative to L‐Kyn treated plants, illustrating the normal response to exogenously applied auxin in the absence of auxin synthesis (Fig. 5d,e). Whilst responses to exogenous auxin in *Ppclv1a1b* and *Ppclv1a1brpk2* mutants were variable between experimental replicates, *Pprpk2* mutants grown on the combined treatment had reduced areas relative to either single treatment (Fig. 5d,e). These data did not fit our hypothesis that *Pprpk2* mutants should respond similarly to exogenously applied auxin regardless of the concentration of endogenous auxin synthesis. Thus, increased auxin responsiveness was insufficient to fully account for the phenotype of *Pprpk2* mutants.

### *Pprpk2* mutants are sensitive to auxin transport inhibition and have low *PIN* expression levels

**Fig. 6:**
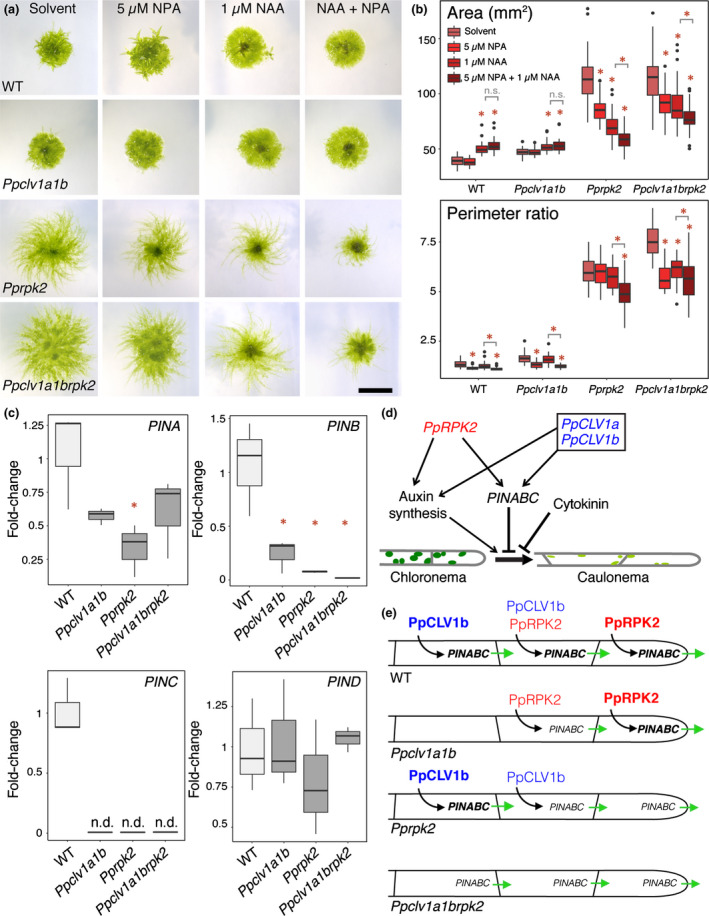
PIN‐mediated auxin transport is dampened in *Physcomitrium patens clavata* mutants. (a) Images of 4‐wk‐old plants grown on a solvent control, 5 µM *N*‐1‐naphthylphthalamic acid (NPA), 1 µM 1‐naphthaleneacetic acid (NAA) or a combination of 5 µM NPA and 1 µM NAA. Bar, 5 mm. (b) Quantitative analyses showed that *Pprpk2* and *Ppclv1a1brpk2* mutant plants showed auxin‐ and auxin transport‐dependent decreases in plant spread and perimeter ratio, whereas wild‐type (WT) and *Ppclv1a1b* plants showed auxin dependent increases in plant spread and transport‐dependent reductions in perimeter ratio (CLV, CLAVATA; RPK, RECEPTOR‐LIKE KINASE). In the boxplot, horizontal lines represent median values, boxes represent the interquartile range, whiskers represent largest and smallest values within 1.5× above or below 75^th^ and 25^th^ percentiles, respectively, and black circles represent outliers. Asterisks above data indicate significant difference from untreated plants of the same genotype, asterisks above bars indicate significant difference between treatments of interest (*n* ≥ 30; one‐way ANOVA and Tukey’s honestly significant difference (HSD) test on each genotype; *P* < 0.05). (c) Quantitative (q)PCR showed that *PINA* expression was depressed in *Pprpk2* mutants relative to WT plants, and *PINB* and *PINC* expression were depressed in all mutants. *PIND* expression showed similar expression levels in WT plants and mutants. *60S* was used as housekeeping control (*n* = 3; n.d., not determined; *, *P* < 0.05 between WT and mutant samples. No significant differences were found between mutants). (d) Model of the CLAVATA gene regulatory network involved in protonemal stem cell identity. Arrows indicate positive regulation and T bars indicate negative regulation. (e) Model of CLAVATA function in WT and *clavata* mutant plants. Black arrows indicate positive regulation of gene expression, green arrows indicate the level and direction of auxin fluxes, and the size and strength of the font reflect the strength of gene action.

The combined L‐Kyn + NAA treatment above would not only change the amount of auxin present, but also its spatial distribution. Therefore, we next hypothesized that local auxin gradients could be important in *Pprpk2* mutant phenotype determination. Promoter fusions have shown that *PIN* auxin transporter genes are highly expressed in protonemal tip cells of *P. patens*, and the chloronemal‐to‐caulonemal transition is accelerated in *pina pinb* mutants but suppressed in *PINA* and *PINB* (and to a lesser extent *PINC*) overexpressors (Viaene *et al*., 2014). To investigate a potential contribution of auxin transport to *Pprpk2* mutant phenotypes, we first took a pharmacological approach, assaying the sensitivity of mutants to transport inhibition. We used naphthylphthalamic acid (NPA) as NPA strongly inhibits PIN function in Arabidopsis and PIN function is conserved (Geldner *et al*., 2001; Abas *et al*., 2021). We grew WT plants, *Ppclv1a1b*, *Pprpk2* and *Ppclv1a1brpk2* mutants on media containing a solvent control, 5 µM NPA, 1 µM NAA, or a combination of 5 µM NPA and 1 µM NAA (Fig. 6a,b). As expected, WT plant spread increased following treatment with auxin in the single and combined treatments, but perimeter ratios decreased following NPA treatment. Whilst *Ppclv1a1b* mutants showed a similar response to WT plants, *Pprpk2* and *Ppclv1a1brpk2* mutants conversely showed mild growth suppression following treatment with 5 µM NPA or 1 µM NAA, and strong growth suppression following the combined treatment. We therefore concluded that *Pprpk2* and *Ppclv1a1brpk2* mutants were hypersensitive to auxin transport inhibition. To assay the molecular basis of sensitivity to auxin transport inhibition, we quantified *PIN* expression in protonemal tissues by qPCR (Fig. 6c). Whilst expression levels of the noncanonical *PIND* did not differ between WT and mutant samples, *PINA* expression was lower in *Pprpk2* mutants and *PINB* expression was lower in all mutants than in WT tissue (Fig. 6c). *PINC* is expressed at around the PCR detection limit in WT protonemata (Bennett *et al*., 2014), and we were unable to detect expression in any mutant. Thus, lower canonical *PIN* expression may contribute to the generation of *Pprpk2* mutant phenotypes by altering the auxin distribution.

## Discussion

The data above led us to a model whereby CLAVATA controls auxin biosynthesis and the expression of *PINA–C*, but *Physcomitrium patens* RECEPTOR‐LIKE KINASE (PpRPK2) has the major role in suppressing the chloronema‐to‐caulonema developmental transition (Fig. 6d). *Pprpk2* and *Ppclv1a1b* mutants show similar reductions in auxin concentrations and *PIN* expression (Figs 4d, 6c), but *Pprpk2* mutants show a much more severe mutant phenotype relative to wild‐type (WT) plants (Fig. 2). As auxin synthesis promotes but auxin transport represses caulonemal development (Ashton *et al*., 1979; Viaene *et al*., 2014; Lavy *et al*., 2016), and CLAVATA promotes auxin synthesis and transport, our data point to a stronger role for auxin transport than synthesis in specifying caulonemal identity. *PpRPK2* is most strongly expressed in tip cells, but *PpCLV1a* and *PpCLV1b* are respectively expressed at low levels or more strongly away from the tip (Fig. 1d). We propose that these differences in gene expression contribute to differences between *Pprpk2* and *Ppclv1a1brpk2* and *Ppclv1a1b* mutant phenotypes, and that *PpRPK2* controls the auxin transport status of tip cells to affect plant growth (Fig. 6e). An altered auxin distribution in the tip cells of *Pprpk2* mutants or subapical cells of *Ppclv1a1b* mutants would support our hypothesis. An alternative hypothesis is that *PpCLV1a* and *PpCLV1b* could act at least partially independently of *PpRPK2*, contributing to observed differences in mutant phenotypes between *Ppcvl1a1b* and *Pprpk2* mutants, and there is some support for this hypothesis in recently published work (Cammarata *et al*., 2021; Demko *et al*., 2021).

Although protonemal tips cells are the distal site of phenotype determination, auxin signalling is concentrated at the centre of plants (proximally) (Menand *et al*., 2007b; Jang & Dolan, 2011). Gain‐of‐function *pACT::PpPINGFP* mutants do not produce caulonemata, and *PIN*s are expressed most strongly in protonemal tip cells and a few subapical cells (Viaene *et al*., 2014). Our model for PpRPK2 function fits with these prior data by suggesting *PIN* expression levels are a primary determinant of protonemal morphology. Protonemal tip cells have low TRANSPORT INHIBITOR RESPONSE (TIR)/AUXIN‐SIGNALLING F‐BOX (AFB)‐mediated auxin sensing (Thelander *et al*., 2019), but can respond strongly to auxin if repressor ARF (PpARFb) levels are low, and PpARFb depletion by tasiRNAs in a subset of filaments patterns caulonema initiation and protonemal branching at plants' foraging fringe (Plavskin *et al*., 2016). *PpCLE* and *PpRPK2* expression are highest in caulonemal tip cells, and as CLE peptides are diffusible, PpRPK2 could act in parallel or in sequence with PpARFb to pattern caulonema initiation (Fig. S7). As CLAVATA genes are not expressed in secondary chloronemata, we propose that differences in the auxin response between chloronemata and caulonemata in WT plants could reflect distinct domains of CLAVATA and *PpARFb* activity.

From an evolutionary perspective, our work suggests that roles for CLAVATA in modulating PIN activity, auxin response and stem cell identity (Pallakies & Simon, 2014; Han & Hwang, 2018; Racolta *et al*., 2018) are conserved within the land plants. In Arabidopsis, the WUSCHEL transcription factor acts downstream of CLAVATA to maintain low auxin conccentrations in stem cells of the central meristem zone. WUSCHEL belongs to the T3 clade of the *WUSCHEL‐LIKE HOMEOBOX* (*WOX*) gene family, and T3 *WOX*es arose from a (T2 + T3) WOX gene duplication pre‐dating the origin of vascular plants (Wu *et al*., 2019). Bryophytes lost the T2/T3 *WOX* precursor lineage (Wu *et al*., 2019), and remaining T1 *WOX* genes act independently of CLAVATA in bryophytes (Sakakibara *et al*., 2014; Hirakawa *et al*., 2020). Thus, it is likely that other transcription factors act in place of WUSCHEL to regulate *PIN* expression in *P*. *patens*. In Arabidopsis, the TDIF/PXY module regulates ARF action independently of *WOX* genes (Han & Hwang, 2018), and we propose that PpRPK2 could act via an ARF intermediary. Our data suggest that downstream components of the CLAVATA gene regulatory network have been remodelled during evolution. WUSCHEL could have been co‐opted into the CLAVATA gene regulatory network in euphyllophytes, or a T3 WUSCHEL precursor could have been co‐opted into the CLAVATA gene regulatory network in the last common ancestor of vascular plants. Alternatively, CLAVATA could have acted via a T2/T3 *WOX* precursor to regulate auxin homeostasis in the last common ancestor of land plants.

## Author contributions

ZNV and JH conceived the project and designed the experiments; AC and YK engineered *promoter::NGG* fusion lines as part of the Leeds Moss Transformation Service; ON and AP performed hormonal quantification and data analysis. All remaining experimental work was performed by ZNV with help from CM, WL and AS and supervision from JH; ZNV and JH analysed the data, wrote the manuscript draft and incorporated feedback from all authors.

## Supporting information


**Fig. S1** Strategy for generation of promoter::NLSGUSGFP reporter lines.
**Fig. S2** *CLAVATA* expression was undetectable WT plants and germinating spores of most *promoter::NLSGUSGFP* reporter lines.
**Fig. S3** *PpcleAmiR1‐3*, *PpcleAmiR4‐7* and *Pprpk2* mutants have subapical cell length and division plane defects in protonemata.
**Fig. S4** *PpcleAmiR1‐3*, *Pprpk2* and *Ppclv1a1brpk2* mutants had protonemal apical dominance defects.
**Fig. S5** *PpcleAmiR* lines have altered levels of *PpCLE1‐9* transcription.
**Fig. S6** No difference in overall cytokinin levels between WT and *clavata* mutant lines was found.
**Fig. S7** Model for activation of caulonemal development with *PpRPK2* repression by *PpARFb*.

**Table S1** List of primers used for *promoter::NGG* line generation.

**Table S2** List of primers used for reverse transcription polymerase chain reaction (RT‐PCR) in *PpcleAmiR* lines (Fig. S5).

**Table S3** List of primers used for quantitative polymerase chain reaction (Q‐PCR).
Please note: Wiley Blackwell are not responsible for the content or functionality of any Supporting Information supplied by the authors. Any queries (other than missing material) should be directed to the *New Phytologist* Central Office.

## Data Availability

Data available on request from the authors.
