# Supplementary material for: CLAVATA modulates auxin homeostasis and transport to regulate stem cell identity and plant shape in a moss
Source: New Phytol. 2022 Feb 8;234(1):149–63. doi: 10.1111/nph.17969 (PMC9303531; doi:10.1111/nph.17969)
Supplement: Supplementary file 1 — Fig. S1 Strategy for generation of promoter::NLSGUSGFP reporter lines. Fig. S2 CLAVATA expression was undetectable WT plants and germinating spores of most promoter::NLSGUSGFP reporter lines. Fig. S3 PpcleAmiR1‐3, PpcleAmiR4‐7 and Pprpk2 mutants have subapical cell length and division plane defects in protonemata. Fig. S4 PpcleAmiR1‐3, Pprpk2 and Ppclv1a1brpk2 mutants had protonemal apical dominance defects. Fig. S5 PpcleAmiR lines have altered levels of PpCLE1‐9 transcription. Fig. S6 No difference in overall cytokinin levels between WT and clavata mutant lines was found. Fig. S7 Model for activation of caulonemal development with PpRPK2 repression by PpARFb. [file NPH-234-149-s001.pdf]

***New Phytologist* Supporting Information**

Article title: *CLAVATA* modulates auxin homeostasis and transport to regulate stem cell identity and plant shape in a moss

Authors: Zoe Nemec-Venza[1], Connor Madden[1,2], Amy Stewart[1], Wei Liu[1], Ondřej Novák[3], Aleš Pěnčík[3], Andrew C. Cuming[4], Yasuko Kamisugi[4] and C. Jill Harrison[1†].

Article acceptance date: 21 December 2021

The following Supporting Information is available for this article:

**Fig. S1** Strategy for generation of *promoter::NLSGUSGFP* reporter lines. (A) Promoter fragments of varying lengths and including 5'UTRs of *PpCLE3*, *PpCLE4*, *PpCLE5* and *PpCLE6* were PCR amplified and cloned into the SmaI site of the PIG1NGGII [39] vector with an NptII casette in place of the BSD cassette. For *PpCLE8* and *PpCLE9* reporter lines, the first few amino acids of the coding sequence were also PCR amplified with promoter fragments. Orange boxes represent promoter fragments, *Nos* represents the *Nos* terminator and NptII represents the *CaMV35S-NptII-CaMVter* resistance cassette used to select positive transformants. Constructs were delivered into plants following linearization with PmeI, and lines were first screened by PCR using PIGF2 and promoter-specific primers or PIGR1 and G6TERMF primers [25] (data not shown). (B) PCR screening was followed by Southern analysis with a vector-specific probe following HindIII or ScaI digestion as indicated in (a). Similar expression patterns were verified in three or more independently generated targeted insertants and lines labelled with asterisks were used in further expression analyses.

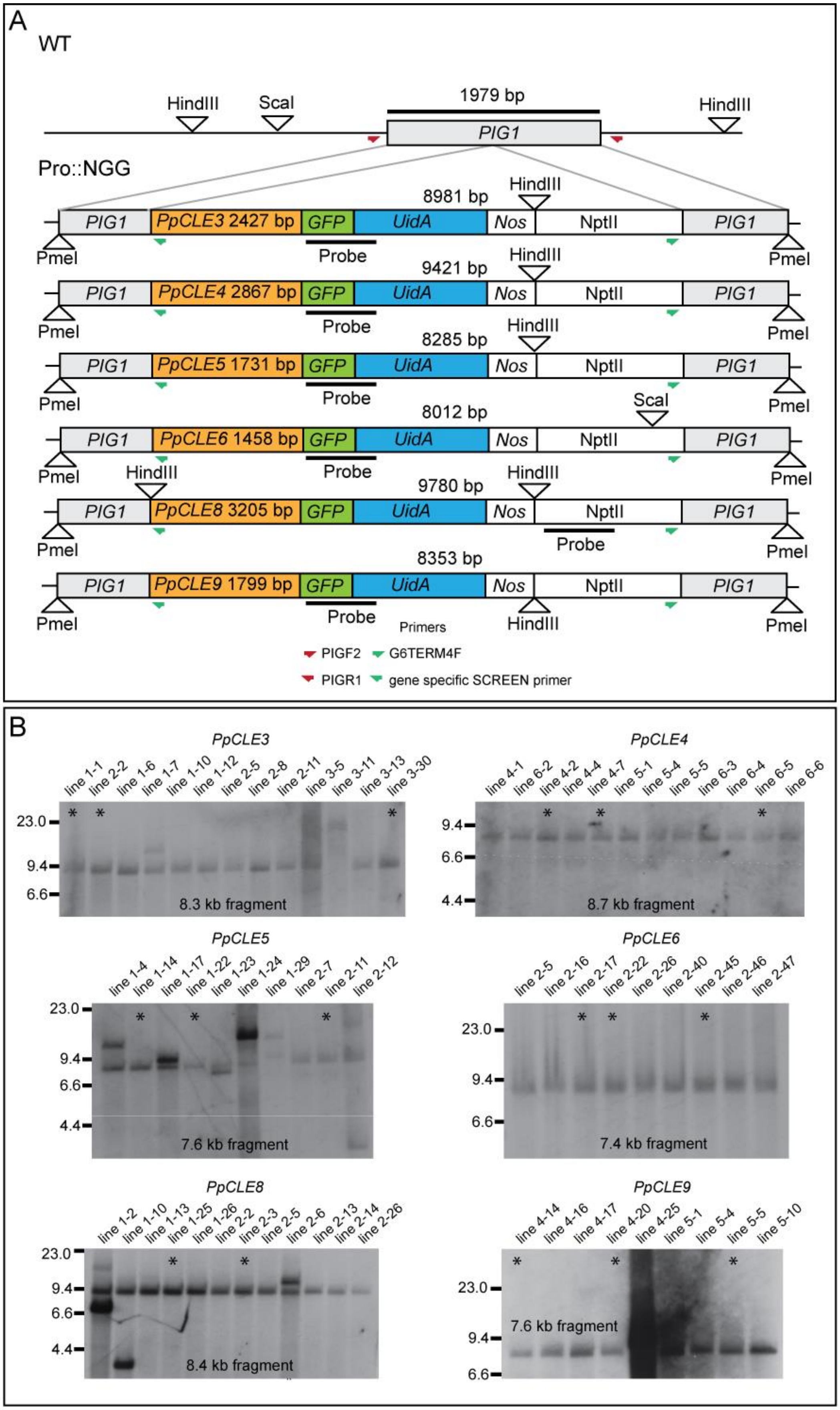
A
WT
HindIII
ScaI
1979 bp
PIG1
HindIII
Pro::NGG
8981 bp
PIG1
PpCLE3 2427 bp
GFP
UidA
Nos
NptII
Probe
PmeI
9421 bp
PpCLE4 2867 bp
8285 bp
PpCLE5 1731 bp
8012 bp
PpCLE6 1458 bp
9780 bp
PpCLE8 3205 bp
8353 bp
PpCLE9 1799 bp
Primers
PIGF2
G6TERM4F
PIGR1
gene specific SCREEN primer
B
PpCLE3
8.3 kb fragment
PpCLE4
8.7 kb fragment
PpCLE5
7.6 kb fragment
PpCLE6
7.4 kb fragment
PpCLE8
8.4 kb fragment
PpCLE9
7.6 kb fragment

**Fig. S2** ***CLAVATA* expression was undetectable wild-type plants and germinating spores of most *promoter::NLSGUSGFP* reporter lines. (A)** Micrographs of germinating GUS-stained spores showing expression only in *PpCLV1a::NGG* tissue. The numbers in each panel indicate the proportion of sporelings displaying a similar expression pattern. Scale bar = 20 µm. **(B,C)** Micrographs of GUS-stained **(B)** wild-type plant and **(C)** filament showing absence of staining in negative controls. Scale bar for (B) = 1 mm, and for (C) = 200 µm.

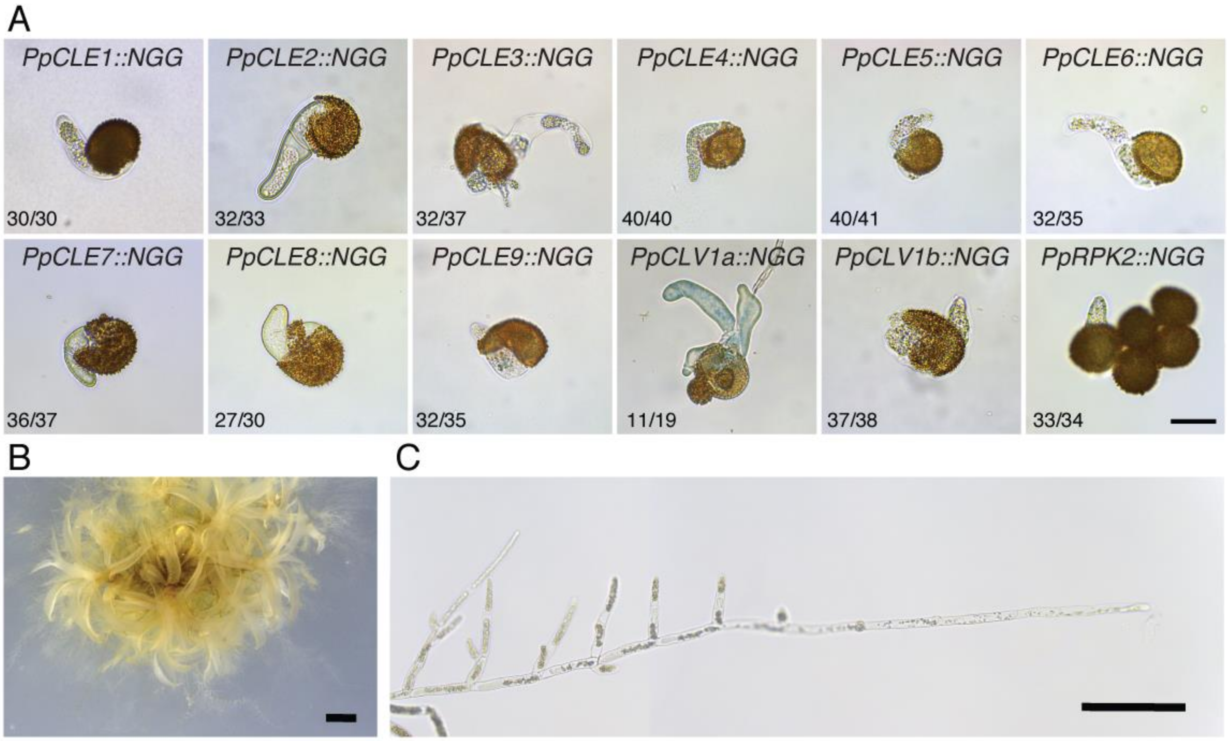

**Fig. S3** ***PpcleAmiR1-3*, *PpcleAmiR4-7* and *Pprpk2* mutants have sub-apical cell length and division plane defects in protonemata. (A)** Analysis of caulonemal sub-apical cell attributes showed that *Pprpk2* cells are longer and with more oblique cell division planes than wild-type cells, *PpcleAmiR1*-3 mutants have divisions that are less oblique and *PpcleAmiR4-7* mutants have longer cells. *Ppclv1a1brpk2* subapical cell measurements are intermediate between wild-type and *Pprpk2* measurements. **(B)** Analysis of chloronemal branch cell attributes showed that *PpcleAmiR1*-3 and *PpcleAmiR4-7* mutants have longer cells than wild-type plants. Bars indicate standard deviation. n ≥ 28, One-way ANOVA and Tukey's test; * = $p < 0.05$.

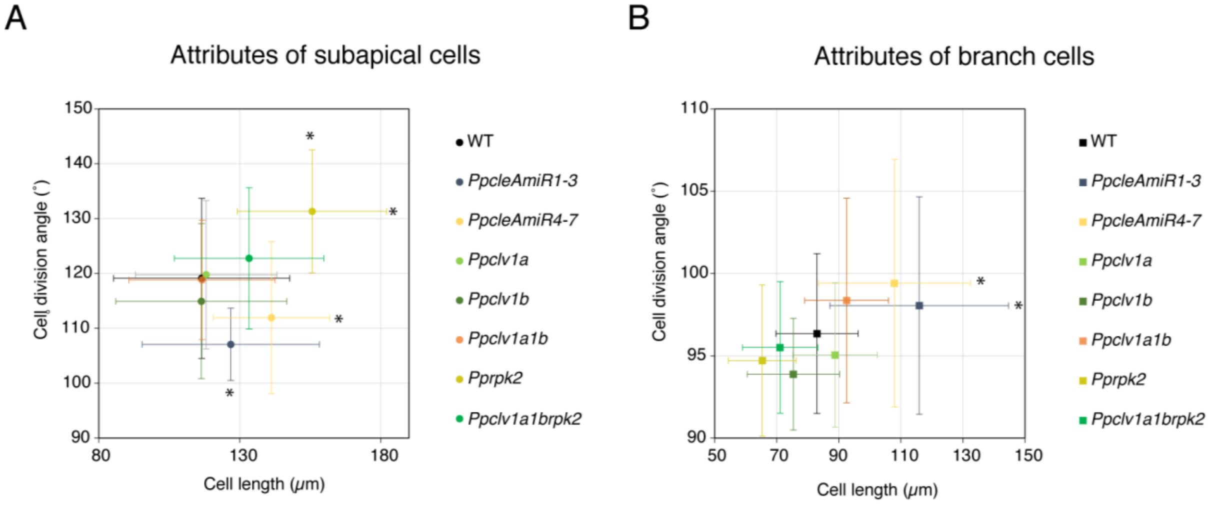

**Fig. S4** ***PpcleAmiR1-3***, ***Pprpk2*** **and** ***Ppclv1a1brpk2*** **mutants had protonemal apical dominance defects.**

*PpcleAmiR1*-3 mutants showed weaker main filament growth relative to side branch growth than wild-type plants and *Pprpk2* and *Ppclv1a1brpk2* mutants showed stronger apical dominance. The length of main filaments from the tip to the base of the first branch with at least 3 cells, and the length of the corresponding branch filament were measured. In the boxplot, horizontal lines represent median values, boxes represent the interquartile range, whiskers represent largest and smallest values within 1.5× above or below 75$^{th}$ and 25$^{th}$ percentiles respectively, and black circles represent outliers. Same lettering indicates no significant between groups, n ≥ 28, one way ANOVA and Tukey's test p = < 0.05.

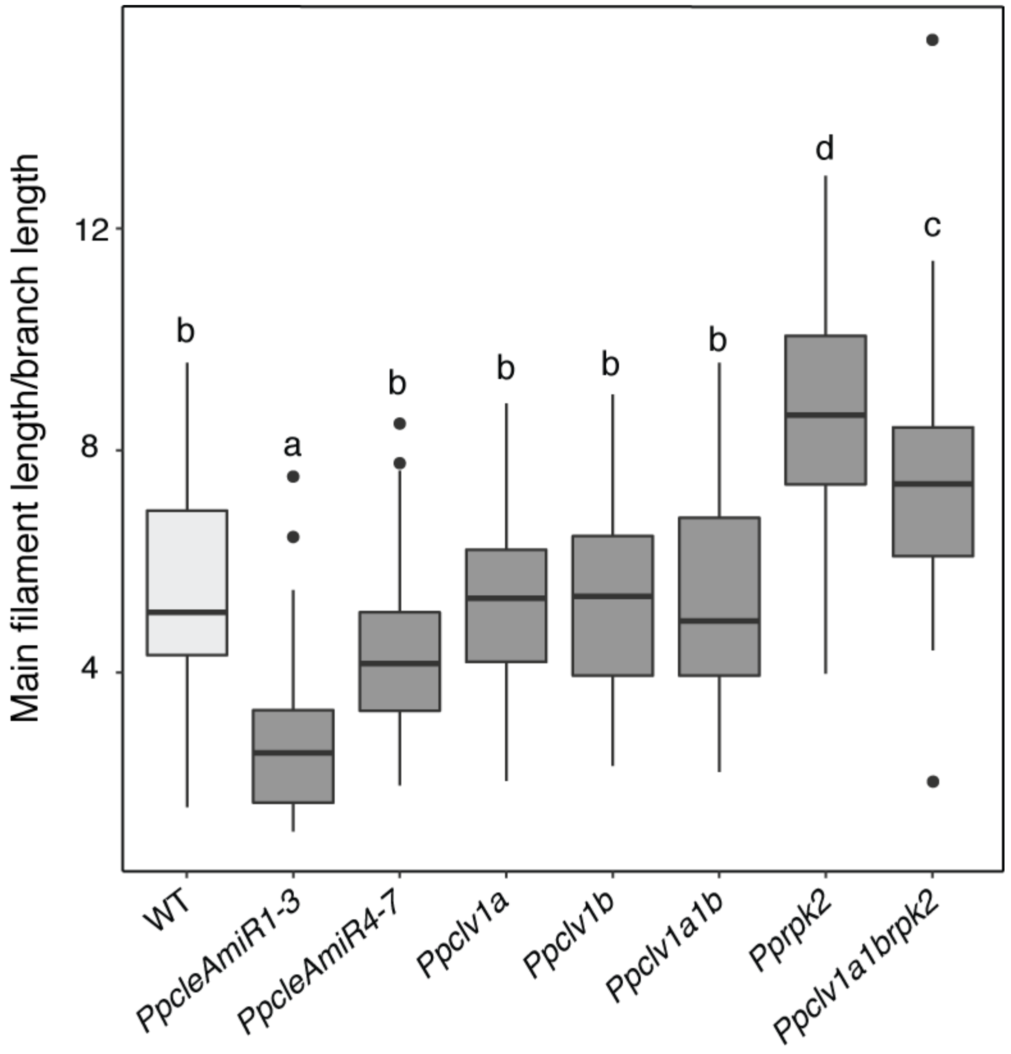

**Fig. S5** ***PpcleAmiR*** **lines have altered levels of** ***PpCLE1-9*** **transcription.** RT-PCR data showing the level of *PpCLE* expression in three independently grown 5-day old protonemal cultures of wild-type plants and *PpcleAmiR1-3* and *PpcleAmiR4-7* mutants. The two bands in the *PpCLE9* lane were sequenced and shown to be splicing variants, with and without an intron. Primer sequences used for amplification are shown in Table S2.

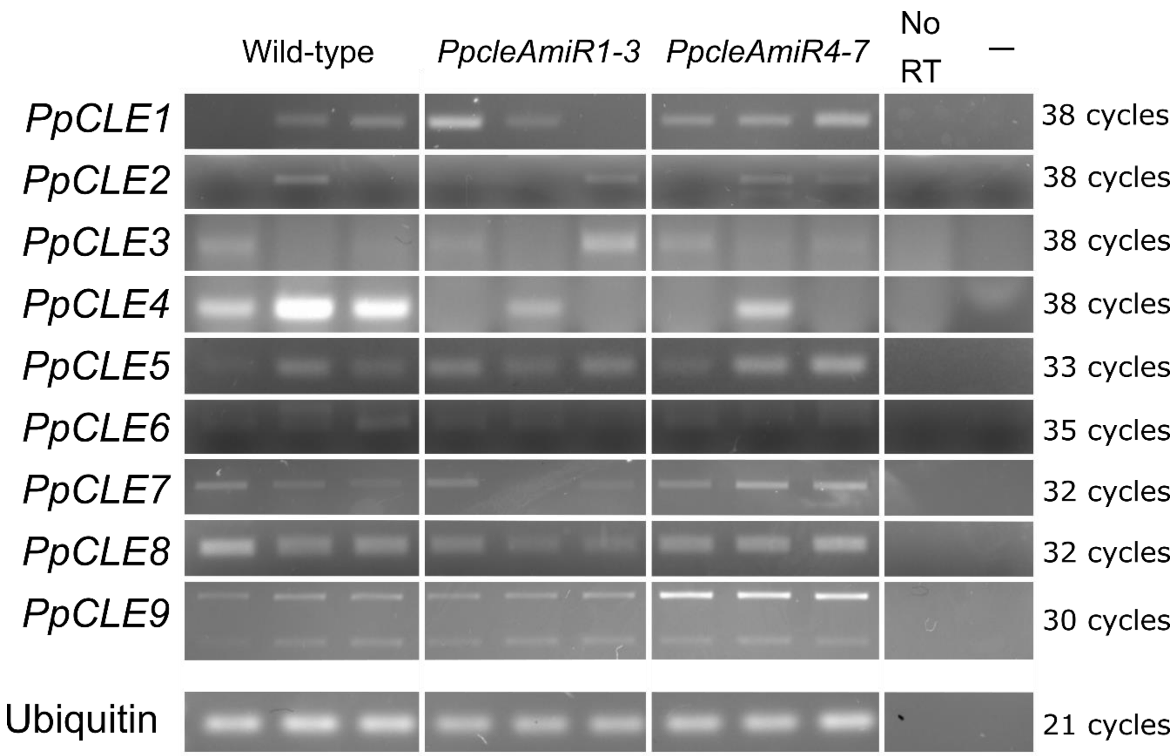

**Fig. S6** No difference in overall cytokinin levels between wild-type and *clavata* mutant lines was found.

While total cytokinin and iP-type cytokinin content was similar between wild-type and mutant plants, cZ content was higher in *Ppclv1a1b* samples and cZRMP content was lower in *Pprpk2* and *Ppclv1a1brpk2* samples. tZOG content was higher in *Ppclv1a1b* and *Pprpk2* mutants than in wild-type plants, with the latter also having higher tZROG. As a result, total tZ-type cytokinins content was higher in *Pprpk2* mutants than in wild-type plants. iP = $N^6$-($\Delta^2$-isopentenyl)adenine; iPR = $N^6$-($\Delta^2$-isopentenyl)adenosine; iPRMP = $N^6$-($\Delta^2$-isopentenyl)adenosine 5′-monophosphate; cZ = cis-zeatin; cZR = cis-zeatin riboside; cZOG = cis-zeatin O-glucoside; cZRMP = cis-zeatin riboside 5′-monophosphate; cZROG = cis-zeatin riboside O-glucoside; tZ = trans-zeatin; tZR = trans-zeatin riboside; tZOG = trans-zeatin O-glucoside; tZROG = trans-zeatin riboside O-glucoside; DHZROG = dihydrozeatin riboside O-glucoside. Other cytokinins were not detected. In the boxplot, horizontal lines represent median values, boxes represent the interquartile range, whiskers represent largest and smallest values within 1.5× above or below 75$^{th}$ and 25$^{th}$ percentiles respectively, and black circles represent outliers. T-test; $n = 5$, * = $p < 0.05$.

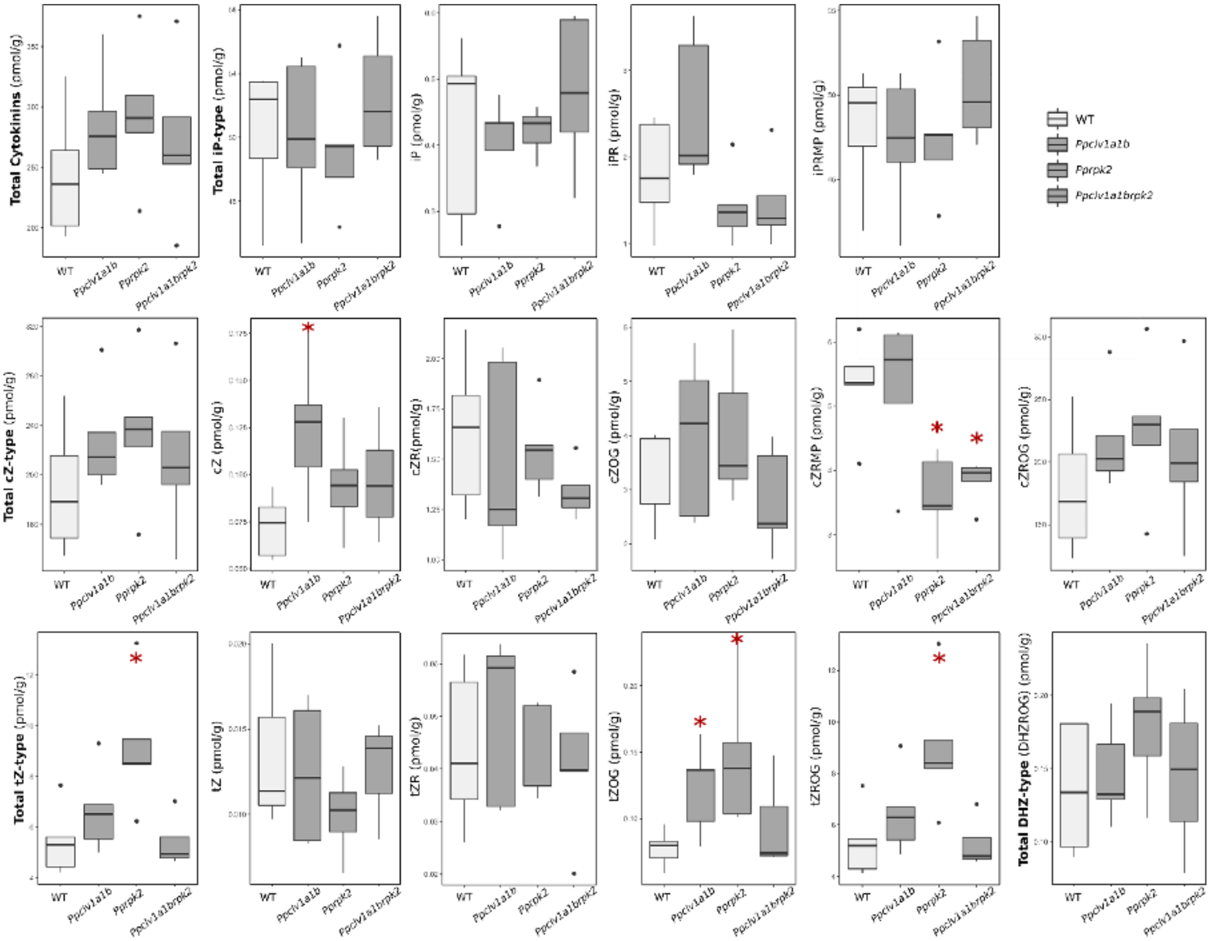

Total Cytokinins (pmol/g)
Total iP-type (pmol/g)
iP (pmol/g)
iPR (pmol/g)
iPRMP (pmol/g)
WT
Ppclv1a1b
Pprpk2
Ppclv1a1brpk2
Total cZ-type (pmol/g)
cZ (pmol/g)
cZR(pmol/g)
cZOG (pmol/g)
cZRMP (pmol/g)
cZROG (pmol/g)
Total tZ-type (pmol/g)
tZ (pmol/g)
tZR (pmol/g)
tZOG (pmol/g)
tZROG (pmol/g)
Total DHZ-type (DHZROG) (pmol/g)

**Fig. S7** Model for activation of caulonemal development with *PpRPK2* repression by *PpARFb*. (A) *PpRPK2* is expressed in few competent chloronemal tip cells lacking PpARFb expression, where it can perceive the presence of neighboring caulonemata or gametophore buds producing CLE peptides. (B) At a later time point, the absence of CLE suppression could enable caulonemal differentiation.

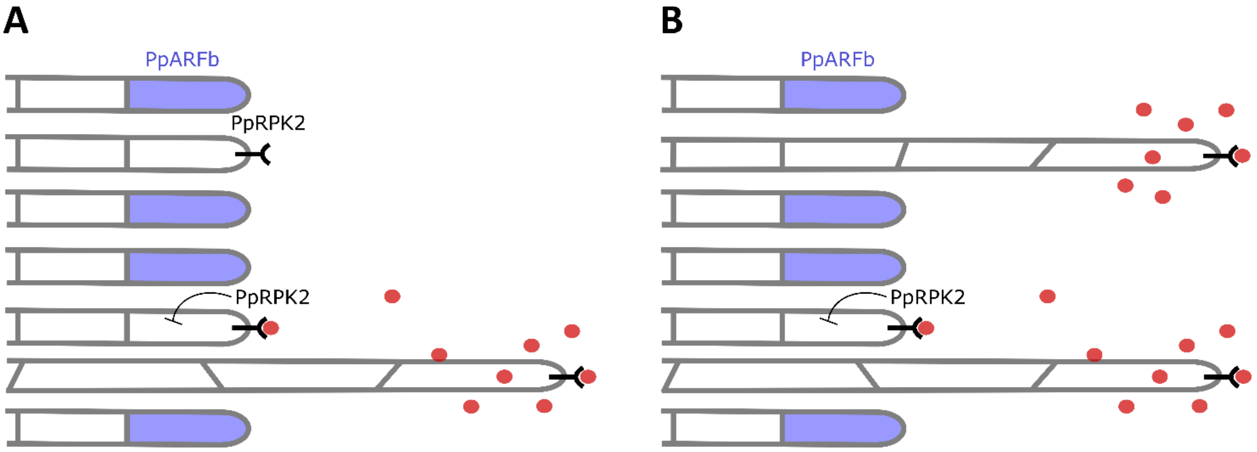

**Table S1** List of primers used for *promoter::NGG* line generation.

**Table S2** List of primers used for RT-PCR in *PpcleAmiR* lines (Figure S5)**.**

**Table S3** List of primers used for Q-PCR.

References from SI tables


Bennett TA, Liu MM, Aoyama T, Bierfreund NM, Braun M, Coudert Y, Dennis RJ, O'Connor D, Wang XY, White CD, et al. 2014. Plasma membrane-targeted PIN proteins drive shoot development in a moss. Current Biology 24: 2776-2785.

Coudert Y, Novák O, Harrison CJ. 2019. A KNOX-cytokinin regulatory module predates the origin of indeterminate vascular plants. Current Biology 29(16): 2743-2750.

Le Bail A, Scholz S, Kost B. 2013. Evaluation of reference genes for RT-qPCR analyses of structure-specific and hormone regulated gene expression in *Physcomitrella patens* gametophytes. PLoS One 8: e70998.

Whitewoods CD, Cammarata J, Nemec Venza Z, Sang S, Crook AD, Aoyama T, Wang XY, Waller M, Kamisugi Y, Cuming AC, et al. 2018. CLAVATA was a genetic novelty for the morphological innovation of 3D growth in land plants. Current Biology 28(15): 2365-2376.
